# [1,1′-Bis(diphenyl­phosphan­yl)ferrocene-κ^2^ *P*,*P*′]dichloridocadmium(II) dichloro­methane disolvate

**DOI:** 10.1107/S1600536810046635

**Published:** 2010-11-17

**Authors:** Chengchen Zhu, Liguo Yang, Dacheng Li

**Affiliations:** a School of Chemistry and Chemical Engineering, Liaocheng University, Shandong 252059, People’s Republic of China

## Abstract

In the title complex, [CdFe(C_17_H_14_P)_2_Cl_2_]·2CH_2_Cl_2_, the Cd^II^ atom has a distorted tetra­hedral coordination geometry by two chloride anions and two P atoms of 1,1′-bis­(diphenyl­phosphan­yl)ferrocene. In the crystal, complex mol­ecules are linked into a three-dimensional network by C—H⋯Cl hydrogen bonds involving the dichloro­methane solvent mol­ecules.

## Related literature

For background to 1,1′-bis­(diphenyl­phosphan­yl)ferrocene metal complexes, see: Corain *et al.* (1989 ▶). For related structures, see: Wang *et al.* (2001 ▶); Huang *et al.* (2002 ▶).
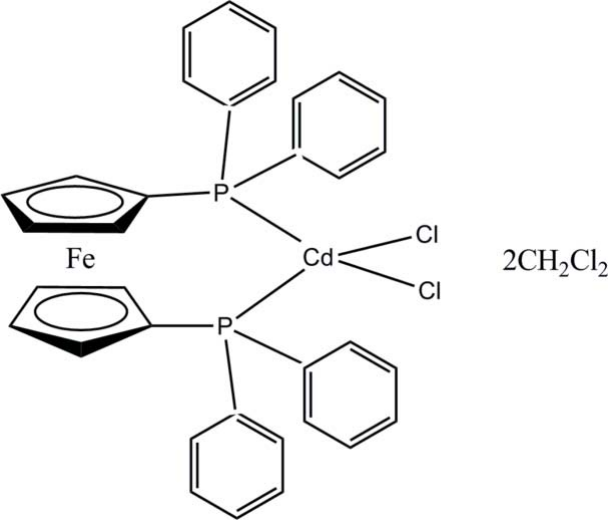


## Experimental

### 

#### Crystal data

[CdFe(C_17_H_14_P)_2_Cl_2_]·2CH_2_Cl_2_
*M* *_r_* = 907.51
Monoclinic, 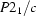

*a* = 9.8114 (10) Å
*b* = 23.594 (2) Å
*c* = 17.6058 (16) Å
β = 93.349 (1)°
*V* = 4068.6 (7) Å^3^
*Z* = 4
Mo *K*α radiation
μ = 1.38 mm^−1^
*T* = 298 K
0.39 × 0.26 × 0.25 mm

#### Data collection

Bruker SMART 1000 CCD diffractometer
Absorption correction: multi-scan (*SADABS*; Siemens, 1996 ▶) *T*_min_ = 0.616, *T*_max_ = 0.725
20307 measured reflections
7133 independent reflections
3982 reflections with *I* > 2σ(*I*)
*R*_int_ = 0.102

#### Refinement

*R*[*F* ^2^ > 2σ(*F* ^2^)] = 0.051
*wR*(*F* ^2^) = 0.106
*S* = 1.00
7133 reflections
415 parameters
H-atom parameters constrained
Δρ_max_ = 0.50 e Å^−3^
Δρ_min_ = −0.71 e Å^−3^

### 

Data collection: *SMART* (Siemens, 1996 ▶); cell refinement: *SAINT* (Siemens, 1996 ▶); data reduction: *SAINT*; program(s) used to solve structure: *SHELXS97* (Sheldrick, 2008 ▶); program(s) used to refine structure: *SHELXL97* (Sheldrick, 2008 ▶); molecular graphics: *SHELXTL* (Sheldrick, 2008 ▶); software used to prepare material for publication: *SHELXTL*.

## Supplementary Material

Crystal structure: contains datablocks I, global. DOI: 10.1107/S1600536810046635/rz2517sup1.cif

Structure factors: contains datablocks I. DOI: 10.1107/S1600536810046635/rz2517Isup2.hkl

Additional supplementary materials:  crystallographic information; 3D view; checkCIF report

## Figures and Tables

**Table 1:** Hydrogen-bond geometry (Å, °)

*D*—H⋯*A*	*D*—H	H⋯*A*	*D*⋯*A*	*D*—H⋯*A*
C35—H35*A*⋯Cl2	0.97	2.77	3.698 (9)	160
C36—H36*B*⋯Cl1	0.97	2.66	3.619 (9)	167
C19—H19⋯Cl2^i^	0.93	2.82	3.746 (7)	173
C27—H27⋯Cl1^ii^	0.93	2.82	3.608 (7)	144

Symmetry codes: (i) 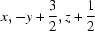; (ii) 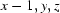.

**Fig. 1.**
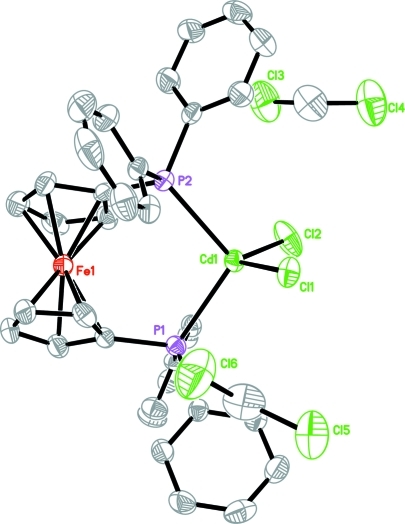
The molecular structure of the title compound, with atom labels and 30% probability displacement ellipsoids. Hydrogen atoms are omitted for clarity.

## supplementary crystallographic information

### Comment

Several efforts have been made to prepare and study metal complexes of 1,1'-bis(diphenylphosphanyl)ferrocene (dppf) not only because of its versatile bonding modes but also for the chemical reactivities of its transition metal complexes (Corain *et al.*, 1989). In order to continue our study in this area, we report here the synthesis and the crystal structure of the title compound.

In the crystal structure of the title compound (Fig. 1), the cadmium(II) atom displays a distorted tetrahedral coordination geometry provided by two chloride anions and two phosphorus donors of dppf. The average Cd—P bond distance of 2.6392 (14) Å is similar to those observed in the literature for related complexes (Wang *et al.*, 2001; Huang *et al.*, 2002). The Cd—Cl bond lengths (mean value 2.4594 (17) Å) are not unexceptional. In the crystal structure, complex molecules and dichloromethane solvent molecules are linked by intemolecular C—H···Cl hydrogen bonds (Table 1) into a three-dimensional network.

### Experimental

[CdCl_2_(dppf)].2CH_2_Cl_2_ was prepared by mixing CdCl_2_.2.5H_2_O (0.22 g, 1 mmol) and dppf (0.55 g, 1 mmol) in 10 mL dichloromethane under the protection of N_2_. The mixture was refluxed for 4 h, then the residue was filtered off and the solution was concentrated to 5 mL. Crystals suitable for X-ray analysis were obtained by slow evaporation of a hexane solution.

### Refinement

All H atoms were placed in geometrically idealized positions (C—H = 0.93–0.97 Å) and treated as riding on their parent atoms, with *U*_iso_(H) = 1.2*U*_eq_(C).

### Crystal data

**Table:**

[CdFe(C_17_H_14_P)_2_Cl_2_]·2CH_2_Cl_2_	*F*(000) = 1816
*M**_r_* = 907.51	*D*_x_ = 1.482 Mg m^−^^3^
Monoclinic, *P*2_1_/*c*	Mo *K*α radiation, λ = 0.71073 Å
Hall symbol: -P 2ybc	Cell parameters from 3906 reflections
*a* = 9.8114 (10) Å	θ = 2.3–21.9°
*b* = 23.594 (2) Å	µ = 1.38 mm^−^^1^
*c* = 17.6058 (16) Å	*T* = 298 K
β = 93.349 (1)°	Block, yellow
*V* = 4068.6 (7) Å^3^	0.39 × 0.26 × 0.25 mm
*Z* = 4	

### Data collection

**Table:**

Bruker SMART 1000 CCD diffractometer	7133 independent reflections
Radiation source: fine-focus sealed tube	3982 reflections with *I* > 2σ(*I*)
graphite	*R*_int_ = 0.102
phi and ω scans	θ_max_ = 25.0°, θ_min_ = 1.4°
Absorption correction: multi-scan (*SADABS*; Siemens, 1996)	*h* = −10→11
*T*_min_ = 0.616, *T*_max_ = 0.725	*k* = −27→28
20307 measured reflections	*l* = −17→20

### Refinement

**Table:**

Refinement on *F*^2^	Primary atom site location: structure-invariant direct methods
Least-squares matrix: full	Secondary atom site location: difference Fourier map
*R*[*F*^2^ > 2σ(*F*^2^)] = 0.051	Hydrogen site location: inferred from neighbouring sites
*wR*(*F*^2^) = 0.106	H-atom parameters constrained
*S* = 1.00	*w* = 1/[σ^2^(*F*_o_^2^) + (0.029*P*)^2^] where *P* = (*F*_o_^2^ + 2*F*_c_^2^)/3
7133 reflections	(Δ/σ)_max_ = 0.001
415 parameters	Δρ_max_ = 0.50 e Å^−^^3^
0 restraints	Δρ_min_ = −0.70 e Å^−^^3^

### Fractional atomic coordinates and isotropic or equivalent isotropic displacement parameters (Å^2^)

**Table:**

	*x*	*y*	*z*	*U*_iso_*/*U*_eq_	
Cd1	0.19344 (4)	0.683033 (18)	0.39863 (2)	0.04745 (14)	
Fe1	−0.09473 (8)	0.64546 (3)	0.56217 (4)	0.0474 (2)	
P1	0.23968 (13)	0.68445 (6)	0.54792 (7)	0.0421 (3)	
P2	−0.06947 (14)	0.66286 (6)	0.37238 (8)	0.0455 (4)	
Cl1	0.22491 (15)	0.77580 (7)	0.33758 (9)	0.0655 (4)	
Cl2	0.3313 (2)	0.60588 (8)	0.34982 (10)	0.0972 (7)	
Cl3	0.1439 (3)	0.45928 (12)	0.24183 (15)	0.1513 (10)	
Cl4	0.3305 (3)	0.50224 (14)	0.13614 (17)	0.1673 (12)	
Cl5	0.3751 (3)	0.93735 (11)	0.43706 (15)	0.1316 (9)	
Cl6	0.0863 (3)	0.92278 (15)	0.46508 (18)	0.1646 (12)	
C1	0.0797 (5)	0.6911 (2)	0.5949 (3)	0.0435 (13)	
C2	0.0318 (6)	0.6598 (3)	0.6586 (3)	0.0574 (16)	
H2	0.0797	0.6321	0.6867	0.069*	
C3	−0.1047 (6)	0.6796 (3)	0.6703 (4)	0.0717 (19)	
H3	−0.1594	0.6669	0.7082	0.086*	
C4	−0.1417 (6)	0.7210 (3)	0.6158 (3)	0.0611 (17)	
H4	−0.2247	0.7401	0.6117	0.073*	
C5	−0.0325 (6)	0.7289 (2)	0.5680 (3)	0.0532 (15)	
H5	−0.0315	0.7537	0.5269	0.064*	
C6	−0.1306 (5)	0.6213 (2)	0.4503 (3)	0.0451 (13)	
C7	−0.0473 (6)	0.5795 (2)	0.4916 (3)	0.0579 (16)	
H7	0.0404	0.5685	0.4805	0.069*	
C8	−0.1233 (8)	0.5578 (3)	0.5524 (3)	0.0714 (19)	
H8	−0.0938	0.5302	0.5872	0.086*	
C9	−0.2511 (8)	0.5859 (3)	0.5502 (4)	0.078 (2)	
H9	−0.3190	0.5798	0.5840	0.094*	
C10	−0.2596 (6)	0.6249 (3)	0.4882 (4)	0.0641 (17)	
H10	−0.3332	0.6483	0.4743	0.077*	
C11	0.3274 (5)	0.6224 (2)	0.5939 (3)	0.0447 (13)	
C12	0.3777 (6)	0.6261 (3)	0.6697 (3)	0.0654 (17)	
H12	0.3718	0.6599	0.6967	0.078*	
C13	0.4378 (7)	0.5775 (3)	0.7045 (4)	0.0747 (19)	
H13	0.4695	0.5791	0.7552	0.090*	
C14	0.4501 (6)	0.5281 (3)	0.6650 (4)	0.0614 (17)	
H14	0.4918	0.4969	0.6887	0.074*	
C15	0.4008 (6)	0.5244 (3)	0.5898 (4)	0.0588 (16)	
H15	0.4078	0.4906	0.5631	0.071*	
C16	0.3403 (5)	0.5720 (2)	0.5543 (3)	0.0523 (15)	
H16	0.3084	0.5698	0.5036	0.063*	
C17	0.3423 (5)	0.7464 (2)	0.5797 (3)	0.0450 (13)	
C18	0.3174 (6)	0.7782 (3)	0.6434 (3)	0.0597 (16)	
H18	0.2443	0.7688	0.6722	0.072*	
C19	0.3998 (7)	0.8241 (3)	0.6649 (4)	0.0743 (19)	
H19	0.3824	0.8446	0.7085	0.089*	
C20	0.5072 (7)	0.8395 (3)	0.6221 (4)	0.078 (2)	
H20	0.5616	0.8704	0.6365	0.094*	
C21	0.5338 (6)	0.8088 (3)	0.5575 (4)	0.0731 (19)	
H21	0.6064	0.8188	0.5286	0.088*	
C22	0.4516 (6)	0.7628 (3)	0.5361 (3)	0.0589 (16)	
H22	0.4690	0.7426	0.4924	0.071*	
C23	−0.1851 (5)	0.7248 (3)	0.3615 (3)	0.0472 (14)	
C24	−0.1393 (6)	0.7791 (3)	0.3794 (3)	0.0580 (16)	
H24	−0.0478	0.7850	0.3942	0.070*	
C25	−0.2296 (9)	0.8251 (3)	0.3753 (4)	0.082 (2)	
H25	−0.2002	0.8614	0.3888	0.098*	
C26	−0.3657 (10)	0.8149 (5)	0.3504 (4)	0.102 (3)	
H26	−0.4275	0.8449	0.3488	0.122*	
C27	−0.4102 (7)	0.7619 (4)	0.3283 (4)	0.089 (2)	
H27	−0.4996	0.7566	0.3090	0.107*	
C28	−0.3209 (6)	0.7167 (3)	0.3349 (4)	0.0736 (19)	
H28	−0.3513	0.6805	0.3215	0.088*	
C29	−0.1060 (5)	0.6230 (2)	0.2843 (3)	0.0480 (14)	
C30	−0.1719 (7)	0.5703 (3)	0.2802 (4)	0.077 (2)	
H30	−0.1981	0.5530	0.3246	0.092*	
C31	−0.1988 (7)	0.5434 (3)	0.2096 (4)	0.081 (2)	
H31	−0.2435	0.5087	0.2075	0.097*	
C32	−0.1598 (7)	0.5681 (3)	0.1444 (4)	0.078 (2)	
H32	−0.1768	0.5497	0.0980	0.094*	
C33	−0.0959 (7)	0.6196 (3)	0.1465 (4)	0.0742 (19)	
H33	−0.0712	0.6363	0.1014	0.089*	
C34	−0.0671 (6)	0.6476 (3)	0.2158 (3)	0.0647 (17)	
H34	−0.0223	0.6823	0.2166	0.078*	
C35	0.1928 (9)	0.5170 (4)	0.1904 (5)	0.122 (3)	
H35A	0.2169	0.5478	0.2250	0.146*	
H35B	0.1163	0.5293	0.1571	0.146*	
C36	0.2509 (9)	0.8924 (4)	0.4712 (5)	0.117 (3)	
H36A	0.2755	0.8831	0.5238	0.140*	
H36B	0.2492	0.8574	0.4422	0.140*	

### Atomic displacement parameters (Å^2^)

**Table:**

	*U*^11^	*U*^22^	*U*^33^	*U*^12^	*U*^13^	*U*^23^
Cd1	0.0418 (3)	0.0547 (3)	0.0464 (2)	0.0029 (2)	0.00698 (17)	−0.0017 (2)
Fe1	0.0460 (5)	0.0483 (5)	0.0486 (5)	−0.0033 (4)	0.0100 (4)	−0.0036 (4)
P1	0.0437 (8)	0.0421 (8)	0.0407 (8)	0.0049 (7)	0.0039 (6)	−0.0022 (7)
P2	0.0382 (9)	0.0512 (9)	0.0472 (8)	−0.0024 (7)	0.0036 (7)	−0.0023 (7)
Cl1	0.0579 (10)	0.0655 (10)	0.0749 (10)	−0.0006 (8)	0.0177 (8)	0.0144 (8)
Cl2	0.1377 (17)	0.0924 (14)	0.0650 (11)	0.0604 (13)	0.0346 (11)	0.0045 (10)
Cl3	0.206 (3)	0.122 (2)	0.131 (2)	0.0052 (19)	0.057 (2)	0.0219 (17)
Cl4	0.178 (3)	0.174 (3)	0.157 (2)	0.051 (2)	0.066 (2)	0.059 (2)
Cl5	0.128 (2)	0.129 (2)	0.143 (2)	0.0139 (16)	0.0478 (16)	0.0320 (17)
Cl6	0.109 (2)	0.192 (3)	0.194 (3)	0.004 (2)	0.0185 (19)	0.043 (2)
C1	0.046 (3)	0.047 (3)	0.038 (3)	0.001 (3)	0.004 (2)	−0.001 (3)
C2	0.064 (4)	0.061 (4)	0.048 (3)	−0.003 (3)	0.011 (3)	−0.001 (3)
C3	0.064 (4)	0.098 (5)	0.056 (4)	−0.015 (4)	0.026 (3)	−0.017 (4)
C4	0.053 (4)	0.066 (4)	0.065 (4)	0.002 (3)	0.013 (3)	−0.015 (4)
C5	0.074 (4)	0.038 (3)	0.048 (3)	0.010 (3)	0.010 (3)	−0.004 (3)
C6	0.035 (3)	0.048 (3)	0.052 (3)	−0.003 (3)	0.001 (3)	−0.010 (3)
C7	0.066 (4)	0.052 (4)	0.058 (4)	−0.002 (3)	0.016 (3)	−0.007 (3)
C8	0.105 (6)	0.052 (4)	0.057 (4)	−0.013 (4)	0.002 (4)	−0.002 (3)
C9	0.083 (6)	0.087 (5)	0.067 (5)	−0.041 (4)	0.018 (4)	0.003 (4)
C10	0.041 (4)	0.076 (5)	0.076 (4)	−0.007 (3)	0.011 (3)	−0.011 (4)
C11	0.043 (3)	0.048 (3)	0.042 (3)	0.003 (3)	−0.003 (3)	−0.003 (3)
C12	0.073 (5)	0.061 (4)	0.061 (4)	0.011 (4)	−0.005 (3)	−0.008 (3)
C13	0.100 (6)	0.064 (5)	0.058 (4)	0.015 (4)	−0.017 (4)	−0.001 (4)
C14	0.056 (4)	0.057 (4)	0.072 (5)	0.007 (3)	0.003 (3)	0.012 (4)
C15	0.061 (4)	0.046 (4)	0.071 (4)	0.002 (3)	0.016 (3)	−0.001 (3)
C16	0.053 (4)	0.052 (4)	0.053 (3)	0.004 (3)	0.009 (3)	−0.003 (3)
C17	0.039 (3)	0.049 (3)	0.047 (3)	0.003 (3)	0.004 (3)	0.004 (3)
C18	0.068 (4)	0.058 (4)	0.054 (4)	−0.007 (3)	0.015 (3)	−0.005 (3)
C19	0.096 (5)	0.063 (5)	0.065 (4)	−0.017 (4)	0.014 (4)	−0.023 (4)
C20	0.080 (5)	0.066 (5)	0.086 (5)	−0.021 (4)	−0.021 (4)	0.005 (4)
C21	0.059 (4)	0.075 (5)	0.086 (5)	−0.013 (4)	0.013 (4)	0.001 (4)
C22	0.050 (4)	0.067 (4)	0.060 (4)	−0.001 (3)	0.007 (3)	−0.007 (3)
C23	0.034 (3)	0.058 (4)	0.049 (3)	0.008 (3)	0.006 (3)	−0.001 (3)
C24	0.052 (4)	0.064 (4)	0.059 (4)	0.008 (4)	0.011 (3)	0.012 (3)
C25	0.096 (6)	0.076 (5)	0.077 (5)	0.029 (5)	0.027 (4)	0.013 (4)
C26	0.106 (8)	0.129 (8)	0.073 (5)	0.077 (7)	0.031 (5)	0.023 (5)
C27	0.050 (5)	0.129 (8)	0.089 (6)	0.029 (5)	0.001 (4)	0.005 (5)
C28	0.044 (4)	0.098 (6)	0.079 (5)	0.013 (4)	0.001 (3)	−0.007 (4)
C29	0.035 (3)	0.052 (4)	0.057 (4)	−0.001 (3)	0.005 (3)	−0.002 (3)
C30	0.090 (5)	0.079 (5)	0.063 (4)	−0.032 (4)	0.017 (4)	−0.005 (4)
C31	0.095 (6)	0.079 (5)	0.069 (5)	−0.035 (4)	0.006 (4)	−0.025 (4)
C32	0.086 (5)	0.084 (6)	0.063 (5)	0.013 (4)	−0.004 (4)	−0.028 (4)
C33	0.101 (6)	0.070 (5)	0.052 (4)	0.005 (4)	0.012 (4)	0.001 (4)
C34	0.069 (4)	0.059 (4)	0.066 (4)	−0.004 (3)	0.005 (3)	0.001 (4)
C35	0.151 (9)	0.090 (6)	0.126 (7)	0.037 (6)	0.015 (6)	−0.001 (6)
C36	0.136 (8)	0.089 (6)	0.123 (7)	0.018 (6)	−0.011 (6)	−0.006 (5)

### Geometric parameters (Å, °)

**Table:**

Cd1—Cl2	2.4530 (17)	C12—H12	0.9300
Cd1—Cl1	2.4658 (16)	C13—C14	1.364 (8)
Cd1—P2	2.6372 (15)	C13—H13	0.9300
Cd1—P1	2.6411 (13)	C14—C15	1.386 (8)
Fe1—C6	2.060 (5)	C14—H14	0.9300
Fe1—C7	2.062 (6)	C15—C16	1.399 (8)
Fe1—C5	2.063 (6)	C15—H15	0.9300
Fe1—C2	2.072 (6)	C16—H16	0.9300
Fe1—C10	2.074 (6)	C17—C18	1.382 (7)
Fe1—C1	2.075 (5)	C17—C22	1.409 (7)
Fe1—C3	2.075 (6)	C18—C19	1.392 (8)
Fe1—C4	2.081 (6)	C18—H18	0.9300
Fe1—C9	2.082 (6)	C19—C20	1.380 (9)
Fe1—C8	2.092 (6)	C19—H19	0.9300
P1—C1	1.823 (5)	C20—C21	1.386 (9)
P1—C17	1.843 (6)	C20—H20	0.9300
P1—C11	1.859 (5)	C21—C22	1.390 (8)
P2—C6	1.817 (6)	C21—H21	0.9300
P2—C29	1.832 (6)	C22—H22	0.9300
P2—C23	1.853 (6)	C23—C24	1.388 (8)
Cl3—C35	1.719 (9)	C23—C28	1.399 (8)
Cl4—C35	1.734 (8)	C24—C25	1.401 (9)
Cl5—C36	1.748 (9)	C24—H24	0.9300
Cl6—C36	1.764 (9)	C25—C26	1.401 (11)
C1—C2	1.446 (7)	C25—H25	0.9300
C1—C5	1.473 (7)	C26—C27	1.373 (11)
C2—C3	1.444 (8)	C26—H26	0.9300
C2—H2	0.9300	C27—C28	1.382 (10)
C3—C4	1.402 (9)	C27—H27	0.9300
C3—H3	0.9300	C28—H28	0.9300
C4—C5	1.413 (7)	C29—C30	1.401 (8)
C4—H4	0.9300	C29—C34	1.410 (7)
C5—H5	0.9300	C30—C31	1.407 (8)
C6—C7	1.450 (8)	C30—H30	0.9300
C6—C10	1.466 (7)	C31—C32	1.362 (9)
C7—C8	1.434 (8)	C31—H31	0.9300
C7—H7	0.9300	C32—C33	1.368 (9)
C8—C9	1.416 (9)	C32—H32	0.9300
C8—H8	0.9300	C33—C34	1.401 (8)
C9—C10	1.428 (9)	C33—H33	0.9300
C9—H9	0.9300	C34—H34	0.9300
C10—H10	0.9300	C35—H35A	0.9700
C11—C16	1.390 (7)	C35—H35B	0.9700
C11—C12	1.398 (7)	C36—H36A	0.9700
C12—C13	1.414 (8)	C36—H36B	0.9700
			
Cl2—Cd1—Cl1	114.86 (6)	Fe1—C7—H7	125.5
Cl2—Cd1—P2	111.00 (7)	C9—C8—C7	108.2 (6)
Cl1—Cd1—P2	103.29 (5)	C9—C8—Fe1	69.8 (4)
Cl2—Cd1—P1	106.97 (5)	C7—C8—Fe1	68.7 (3)
Cl1—Cd1—P1	113.83 (5)	C9—C8—H8	125.9
P2—Cd1—P1	106.62 (4)	C7—C8—H8	125.9
C6—Fe1—C7	41.2 (2)	Fe1—C8—H8	127.2
C6—Fe1—C5	110.2 (2)	C8—C9—C10	109.5 (5)
C7—Fe1—C5	132.4 (2)	C8—C9—Fe1	70.5 (4)
C6—Fe1—C2	152.8 (2)	C10—C9—Fe1	69.6 (3)
C7—Fe1—C2	118.1 (2)	C8—C9—H9	125.2
C5—Fe1—C2	69.0 (2)	C10—C9—H9	125.2
C6—Fe1—C10	41.5 (2)	Fe1—C9—H9	126.2
C7—Fe1—C10	68.9 (2)	C9—C10—C6	107.2 (6)
C5—Fe1—C10	118.2 (3)	C9—C10—Fe1	70.2 (4)
C2—Fe1—C10	163.9 (2)	C6—C10—Fe1	68.7 (3)
C6—Fe1—C1	120.06 (19)	C9—C10—H10	126.4
C7—Fe1—C1	110.3 (2)	C6—C10—H10	126.4
C5—Fe1—C1	41.7 (2)	Fe1—C10—H10	126.2
C2—Fe1—C1	40.8 (2)	C16—C11—C12	119.7 (5)
C10—Fe1—C1	153.6 (2)	C16—C11—P1	120.6 (4)
C6—Fe1—C3	165.9 (2)	C12—C11—P1	119.7 (4)
C7—Fe1—C3	150.5 (3)	C11—C12—C13	118.5 (6)
C5—Fe1—C3	67.2 (3)	C11—C12—H12	120.8
C2—Fe1—C3	40.8 (2)	C13—C12—H12	120.8
C10—Fe1—C3	126.2 (2)	C14—C13—C12	121.3 (6)
C1—Fe1—C3	68.0 (2)	C14—C13—H13	119.3
C6—Fe1—C4	129.8 (2)	C12—C13—H13	119.3
C7—Fe1—C4	169.6 (2)	C13—C14—C15	120.2 (6)
C5—Fe1—C4	39.9 (2)	C13—C14—H14	119.9
C2—Fe1—C4	68.1 (2)	C15—C14—H14	119.9
C10—Fe1—C4	107.4 (2)	C14—C15—C16	119.5 (5)
C1—Fe1—C4	68.3 (2)	C14—C15—H15	120.3
C3—Fe1—C4	39.4 (2)	C16—C15—H15	120.3
C6—Fe1—C9	68.5 (2)	C11—C16—C15	120.7 (5)
C7—Fe1—C9	67.7 (3)	C11—C16—H16	119.6
C5—Fe1—C9	149.7 (3)	C15—C16—H16	119.6
C2—Fe1—C9	126.5 (3)	C18—C17—C22	118.0 (5)
C10—Fe1—C9	40.2 (2)	C18—C17—P1	123.9 (4)
C1—Fe1—C9	165.8 (3)	C22—C17—P1	118.2 (4)
C3—Fe1—C9	106.3 (3)	C17—C18—C19	121.1 (5)
C4—Fe1—C9	116.3 (3)	C17—C18—H18	119.5
C6—Fe1—C8	68.5 (2)	C19—C18—H18	119.5
C7—Fe1—C8	40.4 (2)	C20—C19—C18	120.4 (6)
C5—Fe1—C8	170.2 (3)	C20—C19—H19	119.8
C2—Fe1—C8	107.4 (3)	C18—C19—H19	119.8
C10—Fe1—C8	67.8 (3)	C19—C20—C21	119.8 (6)
C1—Fe1—C8	129.8 (3)	C19—C20—H20	120.1
C3—Fe1—C8	116.5 (3)	C21—C20—H20	120.1
C4—Fe1—C8	148.4 (3)	C20—C21—C22	119.8 (6)
C9—Fe1—C8	39.7 (3)	C20—C21—H21	120.1
C1—P1—C17	105.3 (2)	C22—C21—H21	120.1
C1—P1—C11	105.0 (2)	C21—C22—C17	121.0 (6)
C17—P1—C11	105.1 (2)	C21—C22—H22	119.5
C1—P1—Cd1	110.48 (17)	C17—C22—H22	119.5
C17—P1—Cd1	111.77 (17)	C24—C23—C28	119.5 (6)
C11—P1—Cd1	118.16 (17)	C24—C23—P2	121.1 (4)
C6—P2—C29	107.8 (3)	C28—C23—P2	119.4 (5)
C6—P2—C23	106.0 (2)	C23—C24—C25	120.5 (6)
C29—P2—C23	103.3 (2)	C23—C24—H24	119.7
C6—P2—Cd1	109.14 (18)	C25—C24—H24	119.7
C29—P2—Cd1	112.46 (17)	C24—C25—C26	118.2 (8)
C23—P2—Cd1	117.5 (2)	C24—C25—H25	120.9
C2—C1—C5	106.7 (4)	C26—C25—H25	120.9
C2—C1—P1	129.5 (4)	C27—C26—C25	121.7 (7)
C5—C1—P1	123.6 (4)	C27—C26—H26	119.1
C2—C1—Fe1	69.5 (3)	C25—C26—H26	119.1
C5—C1—Fe1	68.7 (3)	C26—C27—C28	119.3 (8)
P1—C1—Fe1	123.2 (3)	C26—C27—H27	120.3
C3—C2—C1	106.9 (5)	C28—C27—H27	120.3
C3—C2—Fe1	69.8 (3)	C27—C28—C23	120.6 (7)
C1—C2—Fe1	69.7 (3)	C27—C28—H28	119.7
C3—C2—H2	126.5	C23—C28—H28	119.7
C1—C2—H2	126.5	C30—C29—C34	117.9 (5)
Fe1—C2—H2	125.6	C30—C29—P2	124.4 (4)
C4—C3—C2	109.5 (5)	C34—C29—P2	117.6 (4)
C4—C3—Fe1	70.5 (3)	C29—C30—C31	120.5 (6)
C2—C3—Fe1	69.5 (3)	C29—C30—H30	119.8
C4—C3—H3	125.3	C31—C30—H30	119.8
C2—C3—H3	125.3	C32—C31—C30	120.4 (6)
Fe1—C3—H3	126.3	C32—C31—H31	119.8
C3—C4—C5	108.9 (5)	C30—C31—H31	119.8
C3—C4—Fe1	70.1 (4)	C31—C32—C33	120.5 (6)
C5—C4—Fe1	69.4 (3)	C31—C32—H32	119.7
C3—C4—H4	125.5	C33—C32—H32	119.7
C5—C4—H4	125.5	C32—C33—C34	120.7 (6)
Fe1—C4—H4	126.6	C32—C33—H33	119.7
C4—C5—C1	107.9 (5)	C34—C33—H33	119.7
C4—C5—Fe1	70.8 (3)	C33—C34—C29	120.0 (6)
C1—C5—Fe1	69.6 (3)	C33—C34—H34	120.0
C4—C5—H5	126.0	C29—C34—H34	120.0
C1—C5—H5	126.0	Cl3—C35—Cl4	112.5 (4)
Fe1—C5—H5	125.2	Cl3—C35—H35A	109.1
C7—C6—C10	106.8 (5)	Cl4—C35—H35A	109.1
C7—C6—P2	123.0 (4)	Cl3—C35—H35B	109.1
C10—C6—P2	130.0 (5)	Cl4—C35—H35B	109.1
C7—C6—Fe1	69.5 (3)	H35A—C35—H35B	107.8
C10—C6—Fe1	69.7 (3)	Cl5—C36—Cl6	112.8 (5)
P2—C6—Fe1	121.8 (3)	Cl5—C36—H36A	109.0
C8—C7—C6	108.3 (5)	Cl6—C36—H36A	109.0
C8—C7—Fe1	70.9 (3)	Cl5—C36—H36B	109.0
C6—C7—Fe1	69.3 (3)	Cl6—C36—H36B	109.0
C8—C7—H7	125.9	H36A—C36—H36B	107.8
C6—C7—H7	125.9		
			
Cl2—Cd1—P1—C1	−134.47 (19)	C1—Fe1—C6—C10	−155.1 (4)
Cl1—Cd1—P1—C1	97.57 (19)	C3—Fe1—C6—C10	−33.2 (12)
P2—Cd1—P1—C1	−15.65 (19)	C4—Fe1—C6—C10	−69.3 (4)
Cl2—Cd1—P1—C17	108.66 (19)	C9—Fe1—C6—C10	37.5 (4)
Cl1—Cd1—P1—C17	−19.31 (19)	C8—Fe1—C6—C10	80.3 (4)
P2—Cd1—P1—C17	−132.52 (18)	C7—Fe1—C6—P2	−116.9 (5)
Cl2—Cd1—P1—C11	−13.5 (2)	C5—Fe1—C6—P2	15.3 (4)
Cl1—Cd1—P1—C11	−141.51 (19)	C2—Fe1—C6—P2	−67.9 (6)
P2—Cd1—P1—C11	105.27 (19)	C10—Fe1—C6—P2	125.3 (5)
Cl2—Cd1—P2—C6	87.40 (19)	C1—Fe1—C6—P2	−29.8 (4)
Cl1—Cd1—P2—C6	−149.01 (19)	C3—Fe1—C6—P2	92.1 (10)
P1—Cd1—P2—C6	−28.76 (19)	C4—Fe1—C6—P2	56.0 (4)
Cl2—Cd1—P2—C29	−32.2 (2)	C9—Fe1—C6—P2	162.9 (4)
Cl1—Cd1—P2—C29	91.4 (2)	C8—Fe1—C6—P2	−154.4 (4)
P1—Cd1—P2—C29	−148.3 (2)	C10—C6—C7—C8	0.4 (6)
Cl2—Cd1—P2—C23	−151.90 (19)	P2—C6—C7—C8	175.9 (4)
Cl1—Cd1—P2—C23	−28.31 (19)	Fe1—C6—C7—C8	60.5 (4)
P1—Cd1—P2—C23	91.94 (19)	C10—C6—C7—Fe1	−60.1 (4)
C17—P1—C1—C2	−105.5 (5)	P2—C6—C7—Fe1	115.4 (4)
C11—P1—C1—C2	5.2 (6)	C6—Fe1—C7—C8	−119.0 (5)
Cd1—P1—C1—C2	133.7 (5)	C5—Fe1—C7—C8	170.5 (4)
C17—P1—C1—C5	79.6 (5)	C2—Fe1—C7—C8	84.0 (4)
C11—P1—C1—C5	−169.7 (4)	C10—Fe1—C7—C8	−80.1 (4)
Cd1—P1—C1—C5	−41.2 (5)	C1—Fe1—C7—C8	128.2 (4)
C17—P1—C1—Fe1	164.7 (3)	C3—Fe1—C7—C8	47.1 (7)
C11—P1—C1—Fe1	−84.6 (3)	C4—Fe1—C7—C8	−151.2 (12)
Cd1—P1—C1—Fe1	43.9 (3)	C9—Fe1—C7—C8	−36.7 (4)
C6—Fe1—C1—C2	−154.4 (3)	C5—Fe1—C7—C6	−70.5 (4)
C7—Fe1—C1—C2	−109.9 (3)	C2—Fe1—C7—C6	−157.0 (3)
C5—Fe1—C1—C2	118.4 (4)	C10—Fe1—C7—C6	38.9 (3)
C10—Fe1—C1—C2	166.8 (5)	C1—Fe1—C7—C6	−112.8 (3)
C3—Fe1—C1—C2	38.5 (4)	C3—Fe1—C7—C6	166.1 (4)
C4—Fe1—C1—C2	81.1 (4)	C4—Fe1—C7—C6	−32.2 (15)
C9—Fe1—C1—C2	−30.6 (11)	C9—Fe1—C7—C6	82.3 (4)
C8—Fe1—C1—C2	−68.4 (4)	C8—Fe1—C7—C6	119.0 (5)
C6—Fe1—C1—C5	87.2 (3)	C6—C7—C8—C9	−0.7 (7)
C7—Fe1—C1—C5	131.7 (3)	Fe1—C7—C8—C9	58.8 (4)
C2—Fe1—C1—C5	−118.4 (4)	C6—C7—C8—Fe1	−59.5 (4)
C10—Fe1—C1—C5	48.3 (6)	C6—Fe1—C8—C9	−81.8 (4)
C3—Fe1—C1—C5	−79.9 (4)	C7—Fe1—C8—C9	−120.0 (5)
C4—Fe1—C1—C5	−37.3 (3)	C2—Fe1—C8—C9	126.8 (4)
C9—Fe1—C1—C5	−149.0 (10)	C10—Fe1—C8—C9	−36.9 (4)
C8—Fe1—C1—C5	173.2 (3)	C1—Fe1—C8—C9	166.4 (3)
C6—Fe1—C1—P1	−29.9 (4)	C3—Fe1—C8—C9	83.7 (4)
C7—Fe1—C1—P1	14.6 (4)	C4—Fe1—C8—C9	50.5 (7)
C5—Fe1—C1—P1	−117.1 (4)	C6—Fe1—C8—C7	38.2 (3)
C2—Fe1—C1—P1	124.5 (5)	C2—Fe1—C8—C7	−113.2 (4)
C10—Fe1—C1—P1	−68.8 (6)	C10—Fe1—C8—C7	83.2 (4)
C3—Fe1—C1—P1	163.0 (4)	C1—Fe1—C8—C7	−73.6 (4)
C4—Fe1—C1—P1	−154.4 (4)	C3—Fe1—C8—C7	−156.3 (4)
C9—Fe1—C1—P1	93.9 (10)	C4—Fe1—C8—C7	170.5 (4)
C8—Fe1—C1—P1	56.1 (4)	C9—Fe1—C8—C7	120.0 (6)
C5—C1—C2—C3	−1.3 (6)	C7—C8—C9—C10	0.7 (7)
P1—C1—C2—C3	−176.8 (4)	Fe1—C8—C9—C10	58.8 (5)
Fe1—C1—C2—C3	−60.1 (4)	C7—C8—C9—Fe1	−58.1 (4)
C5—C1—C2—Fe1	58.8 (3)	C6—Fe1—C9—C8	81.9 (4)
P1—C1—C2—Fe1	−116.7 (4)	C7—Fe1—C9—C8	37.3 (4)
C6—Fe1—C2—C3	172.7 (5)	C5—Fe1—C9—C8	175.3 (4)
C7—Fe1—C2—C3	−153.1 (4)	C2—Fe1—C9—C8	−72.0 (5)
C5—Fe1—C2—C3	79.0 (4)	C10—Fe1—C9—C8	120.6 (5)
C10—Fe1—C2—C3	−40.6 (11)	C1—Fe1—C9—C8	−47.5 (11)
C1—Fe1—C2—C3	117.8 (5)	C3—Fe1—C9—C8	−112.0 (4)
C4—Fe1—C2—C3	36.1 (4)	C4—Fe1—C9—C8	−153.2 (4)
C9—Fe1—C2—C3	−71.1 (5)	C6—Fe1—C9—C10	−38.8 (4)
C8—Fe1—C2—C3	−110.6 (4)	C7—Fe1—C9—C10	−83.3 (4)
C6—Fe1—C2—C1	54.8 (6)	C5—Fe1—C9—C10	54.7 (6)
C7—Fe1—C2—C1	89.1 (4)	C2—Fe1—C9—C10	167.4 (3)
C5—Fe1—C2—C1	−38.8 (3)	C1—Fe1—C9—C10	−168.1 (8)
C10—Fe1—C2—C1	−158.4 (8)	C3—Fe1—C9—C10	127.3 (4)
C3—Fe1—C2—C1	−117.8 (5)	C4—Fe1—C9—C10	86.2 (4)
C4—Fe1—C2—C1	−81.8 (3)	C8—Fe1—C9—C10	−120.6 (5)
C9—Fe1—C2—C1	171.0 (4)	C8—C9—C10—C6	−0.4 (7)
C8—Fe1—C2—C1	131.5 (3)	Fe1—C9—C10—C6	59.0 (4)
C1—C2—C3—C4	0.8 (7)	C8—C9—C10—Fe1	−59.4 (5)
Fe1—C2—C3—C4	−59.3 (4)	C7—C6—C10—C9	0.0 (6)
C1—C2—C3—Fe1	60.1 (4)	P2—C6—C10—C9	−175.0 (4)
C6—Fe1—C3—C4	−45.5 (12)	Fe1—C6—C10—C9	−59.9 (4)
C7—Fe1—C3—C4	174.9 (4)	C7—C6—C10—Fe1	59.9 (4)
C5—Fe1—C3—C4	36.9 (4)	P2—C6—C10—Fe1	−115.1 (5)
C2—Fe1—C3—C4	120.7 (5)	C6—Fe1—C10—C9	118.6 (6)
C10—Fe1—C3—C4	−72.2 (5)	C7—Fe1—C10—C9	80.0 (4)
C1—Fe1—C3—C4	82.1 (4)	C5—Fe1—C10—C9	−152.2 (4)
C9—Fe1—C3—C4	−111.7 (4)	C2—Fe1—C10—C9	−39.2 (11)
C8—Fe1—C3—C4	−153.1 (4)	C1—Fe1—C10—C9	173.5 (5)
C6—Fe1—C3—C2	−166.2 (9)	C3—Fe1—C10—C9	−71.0 (5)
C7—Fe1—C3—C2	54.2 (6)	C4—Fe1—C10—C9	−110.3 (4)
C5—Fe1—C3—C2	−83.8 (4)	C8—Fe1—C10—C9	36.4 (4)
C10—Fe1—C3—C2	167.1 (4)	C7—Fe1—C10—C6	−38.6 (3)
C1—Fe1—C3—C2	−38.5 (3)	C5—Fe1—C10—C6	89.2 (4)
C4—Fe1—C3—C2	−120.7 (5)	C2—Fe1—C10—C6	−157.8 (8)
C9—Fe1—C3—C2	127.6 (4)	C1—Fe1—C10—C6	54.9 (6)
C8—Fe1—C3—C2	86.2 (4)	C3—Fe1—C10—C6	170.5 (4)
C2—C3—C4—C5	0.1 (7)	C4—Fe1—C10—C6	131.1 (4)
Fe1—C3—C4—C5	−58.6 (4)	C9—Fe1—C10—C6	−118.6 (6)
C2—C3—C4—Fe1	58.7 (4)	C8—Fe1—C10—C6	−82.2 (4)
C6—Fe1—C4—C3	166.9 (3)	C1—P1—C11—C16	109.4 (4)
C7—Fe1—C4—C3	−165.9 (12)	C17—P1—C11—C16	−139.8 (4)
C5—Fe1—C4—C3	−120.3 (5)	Cd1—P1—C11—C16	−14.3 (5)
C2—Fe1—C4—C3	−37.3 (3)	C1—P1—C11—C12	−69.0 (5)
C10—Fe1—C4—C3	126.3 (4)	C17—P1—C11—C12	41.8 (5)
C1—Fe1—C4—C3	−81.4 (4)	Cd1—P1—C11—C12	167.4 (4)
C9—Fe1—C4—C3	83.9 (4)	C16—C11—C12—C13	−1.5 (9)
C8—Fe1—C4—C3	50.6 (7)	P1—C11—C12—C13	176.8 (5)
C6—Fe1—C4—C5	−72.8 (4)	C11—C12—C13—C14	1.6 (10)
C7—Fe1—C4—C5	−45.6 (15)	C12—C13—C14—C15	−1.4 (10)
C2—Fe1—C4—C5	83.1 (4)	C13—C14—C15—C16	1.1 (9)
C10—Fe1—C4—C5	−113.3 (4)	C12—C11—C16—C15	1.2 (8)
C1—Fe1—C4—C5	39.0 (3)	P1—C11—C16—C15	−177.1 (4)
C3—Fe1—C4—C5	120.3 (5)	C14—C15—C16—C11	−1.0 (8)
C9—Fe1—C4—C5	−155.8 (4)	C1—P1—C17—C18	18.7 (5)
C8—Fe1—C4—C5	170.9 (5)	C11—P1—C17—C18	−92.0 (5)
C3—C4—C5—C1	−0.9 (6)	Cd1—P1—C17—C18	138.6 (4)
Fe1—C4—C5—C1	−59.9 (4)	C1—P1—C17—C22	−160.6 (4)
C3—C4—C5—Fe1	59.1 (4)	C11—P1—C17—C22	88.8 (5)
C2—C1—C5—C4	1.3 (6)	Cd1—P1—C17—C22	−40.6 (5)
P1—C1—C5—C4	177.2 (4)	C22—C17—C18—C19	−1.6 (9)
Fe1—C1—C5—C4	60.7 (4)	P1—C17—C18—C19	179.1 (5)
C2—C1—C5—Fe1	−59.3 (4)	C17—C18—C19—C20	1.2 (10)
P1—C1—C5—Fe1	116.5 (4)	C18—C19—C20—C21	−0.5 (10)
C6—Fe1—C5—C4	128.6 (4)	C19—C20—C21—C22	0.4 (10)
C7—Fe1—C5—C4	170.0 (4)	C20—C21—C22—C17	−0.9 (10)
C2—Fe1—C5—C4	−80.5 (4)	C18—C17—C22—C21	1.5 (9)
C10—Fe1—C5—C4	83.6 (4)	P1—C17—C22—C21	−179.2 (5)
C1—Fe1—C5—C4	−118.5 (5)	C6—P2—C23—C24	111.5 (5)
C3—Fe1—C5—C4	−36.5 (4)	C29—P2—C23—C24	−135.3 (5)
C9—Fe1—C5—C4	46.9 (6)	Cd1—P2—C23—C24	−10.8 (5)
C6—Fe1—C5—C1	−112.9 (3)	C6—P2—C23—C28	−68.6 (5)
C7—Fe1—C5—C1	−71.5 (4)	C29—P2—C23—C28	44.7 (5)
C2—Fe1—C5—C1	38.0 (3)	Cd1—P2—C23—C28	169.1 (4)
C10—Fe1—C5—C1	−157.9 (3)	C28—C23—C24—C25	3.9 (8)
C3—Fe1—C5—C1	82.0 (3)	P2—C23—C24—C25	−176.1 (4)
C4—Fe1—C5—C1	118.5 (5)	C23—C24—C25—C26	−2.1 (9)
C9—Fe1—C5—C1	165.5 (4)	C24—C25—C26—C27	−1.8 (10)
C29—P2—C6—C7	88.9 (5)	C25—C26—C27—C28	3.9 (11)
C23—P2—C6—C7	−161.0 (4)	C26—C27—C28—C23	−2.0 (10)
Cd1—P2—C6—C7	−33.5 (5)	C24—C23—C28—C27	−1.8 (9)
C29—P2—C6—C10	−96.8 (5)	P2—C23—C28—C27	178.2 (5)
C23—P2—C6—C10	13.2 (6)	C6—P2—C29—C30	1.0 (6)
Cd1—P2—C6—C10	140.7 (5)	C23—P2—C29—C30	−110.9 (6)
C29—P2—C6—Fe1	173.8 (3)	Cd1—P2—C29—C30	121.4 (5)
C23—P2—C6—Fe1	−76.1 (4)	C6—P2—C29—C34	179.9 (4)
Cd1—P2—C6—Fe1	51.4 (3)	C23—P2—C29—C34	67.9 (5)
C5—Fe1—C6—C7	132.1 (3)	Cd1—P2—C29—C34	−59.8 (5)
C2—Fe1—C6—C7	48.9 (6)	C34—C29—C30—C31	−0.5 (10)
C10—Fe1—C6—C7	−117.8 (5)	P2—C29—C30—C31	178.4 (5)
C1—Fe1—C6—C7	87.0 (4)	C29—C30—C31—C32	0.6 (11)
C3—Fe1—C6—C7	−151.0 (10)	C30—C31—C32—C33	−1.0 (11)
C4—Fe1—C6—C7	172.8 (3)	C31—C32—C33—C34	1.1 (11)
C9—Fe1—C6—C7	−80.3 (4)	C32—C33—C34—C29	−1.0 (10)
C8—Fe1—C6—C7	−37.5 (4)	C30—C29—C34—C33	0.7 (9)
C7—Fe1—C6—C10	117.8 (5)	P2—C29—C34—C33	−178.3 (5)
C5—Fe1—C6—C10	−110.1 (4)		

### Hydrogen-bond geometry (Å, °)

**Table:**

*D*—H···*A*	*D*—H	H···*A*	*D*···*A*	*D*—H···*A*
C35—H35A···Cl2	0.97	2.77	3.698 (9)	160
C36—H36B···Cl1	0.97	2.66	3.619 (9)	167
C19—H19···Cl2^i^	0.93	2.82	3.746 (7)	173
C27—H27···Cl1^ii^	0.93	2.82	3.608 (7)	144

Symmetry codes: (i) *x*, −*y*+3/2, *z*+1/2; (ii) *x*−1, *y*, *z*.
